# Modeling and systematic analysis of biomarker validation using selected reaction monitoring

**DOI:** 10.1186/s13637-014-0017-y

**Published:** 2014-11-15

**Authors:** Esmaeil Atashpaz-Gargari, Ulisses M Braga-Neto, Edward R Dougherty

**Affiliations:** 1 Department of Electrical and Computer Engineering, Texas A&M University, 3128 TAMU, College Station, 77843-3128 TX USA; 2 Center for Bionformatics and Genomic Systems Engineering, Texas A&M University, 101 Gateway Blvd, College Station, 77845 TX USA

**Keywords:** Proteomics, Biomarker validation, Mass spectrometry (MS), Selected reaction monitoring (SRM), Triple quadrupole (QQQ) systems

## Abstract

**Background:**

Discovery and validation of protein biomarkers with high specificity is the main challenge of current proteomics studies. Different mass spectrometry models are used as shotgun tools for the discovery of biomarkers. Validation of a set of selected biomarkers from a list of candidates is an important stage in the biomarker identification pipeline. Validation is typically done by triple quadrupole (QQQ) mass spectrometry (MS) running in selected reaction monitoring (SRM) mode. Although the individual modules of this pipeline have been studied, there is little work on integrating the components from a systematic point of view.

**Results:**

This paper analyzes the SRM experiment pipeline in a systematic fashion, by modeling the main stages of the biomarker validation process. The proposed models for SRM and protein mixture are then used to study the effect of different parameters on the final performance of biomarker validation. Sample complexity, purification, peptide ionization, and peptide specificity are among the parameters of the SRM experiment that are studied. We focus on the sensitivity of the SRM pipeline to the working parameters, in order to identify the bottlenecks where time and energy should be spent in designing the experiment.

**Conclusions:**

The model presented in this paper can be utilized to observe the effect of different instrument and experimental settings on biomarker validation by SRM. On the other hand, the model would be beneficial for optimization of the work flow as well as identification of the bottlenecks of the pipeline. Also, it creates the required infrastructure for predicting the performance of the SRM pipeline for a specific setting of the parameters.

**Electronic supplementary material:**

The online version of this article (doi:10.1186/s13637-014-0017-y) contains supplementary material, which is available to authorized users.

## 1 Introduction

### 1.1 Proteomics and mass spectrometry

Proteomics deals with the study of gene and cellular function at the protein level. Microarrays, 2D gel electrophoresis, and mass spectrometry (MS) are the most widely used technologies for high-throughput proteomics. Among these technologies, MS has increasingly become the method of the choice for analysis of complex protein samples [1]. Among its unique advantages are unsurpassed molecular specificity and very high detection sensitivity [2]. MS analysis is composed of thee major steps: 1) *ionization*: conversion of the analyte molecules or atoms into gas-phase ionic species, 2) *mass analysis*: separation and mass analysis of ions on the basis of their mass-to-charge (*m*/*z*) ratio, and 3) *detection*: detection and measurement of the mass-separated ions.

Time of flight (TOF), linear quadrupole/3D-quadrupole ion trap, Fourier transform ion cyclotron resonance (FT-ICR), and orbitrap are some of the main mass analyzers used in MS instruments. Application of two or more stages of mass analysis leads to tandem mass spectrometry (MS/MS) which enables us to examine selectively the fragmentation of particular ions in a mixture of ions [3]. Selected reaction monitoring (SRM) is a specific mode of tandem mass spectrometry, which is widely used for quantitative measurement of analytes present in complex mixture and for validation of low-abundance biomarkers.

### 1.2 Biomarker discovery and validation

The identification of biomarkers is a major goal of biomedicine in this century [4], and proteomics using different mass spectrometry tools has played a key role in this area. One well-known example of peptide biomarker is prostate-specific antigen (PSA), which is a marker for early diagnosis of prostate cancer in men. The PSA test is an FDA-approved serum or plasma-based population screening tool but has very low specificity, resulting in $750 million annual cost for unnecessary medical follow-up. The lack of biomarkers with high specificity shows how challenging the problem of proteomic biomarker identification is and the need for sensitive and accurate instruments, powerful techniques, and careful analysis of proteomics data.

**Figure 1:**
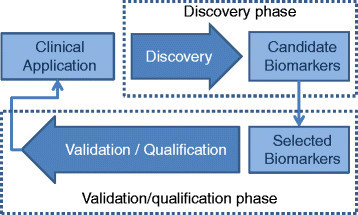
**Two main stages of biomarker development pipeline.** The discovery phase requires MS experiments with high resolution and short duty cycles and typically involves small number of samples. Selected biomarkers from the discovery step are validated in the next stage before moving on to further analysis in clinical studies [4].

One of the important challenges of biomarker discovery is identification of low-abundance biomarkers. Abundant biomarkers are easy to detect and quantify, but these have already been identified for the most part. The current emphasis is therefore on the discovery of low-abundance biomarkers [4]. Figure 1 displays the biomarker identification pipeline and the two main stages in this process, the *discovery* and *validation/qualification* phases. The global discovery phase is done on a small number of samples, and then a larger number of samples is used for the validation of potential biomarkers, before going to clinical application [4].

### 1.3 Selected reaction monitoring

For over 30 years, SRM has been the method of choice for doing mass spectrometry on small molecules in order to study drug metabolism. However, its application to protein identification and quantification was limited by the low mass range of the instruments used for metabolite identification. The introduction of the quadrupole instrument with extended mass range removed this restriction in the application of SRM for studying proteins and peptides [4]-[6]. Although SRM can be done on some of the other tandem MS instruments (e.g., EB- and BE-magnetic sector tandem MS), it is preferably implemented on triple-quadrupole, due to low cost, linear mass scale, operational simplicity, and straightforward scan laws. The first and third quadrupoles in the triple quadrupole (QQQ) systems act as mass filters to specifically select a predefined *m*/*z* values, controlled by direct current (dc) and radio frequency (rf) potentials. The second quadrupole in SRM operates as rf-only quadrupole passing all ions. In fact, this quadrupole acts as the collision-induced dissociation (CID) unit. This is done in two steps: *collision activation* and *collisionally activated dissociation* and is performed in the *high-* and *low-energy* regimes. The later is the mode that is preferably implemented in quadrupole. One of the main disadvantages of CID over other ion activation and dissociation methods is that ion-dissociation efficiency gradually falls off as the precursor ion’s weight increases.

**Figure 2:**
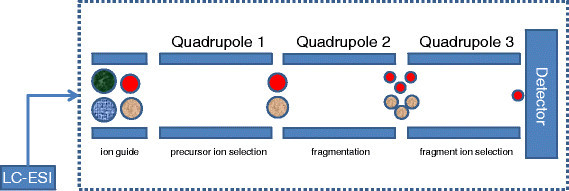
**Idealized schematics of QQQ MS used in SRM analysis.** The first quadruple (Q1) filters out most co-eluting ions from the chromatographic system. However, interfering ions may pass Q1 and enter the second quadruple (Q2). Ions in Q2 are fragmented and form the input of the third quadruple (Q3). Ideally, the specific *m*/*z* selection in Q3 passes only fragments of the desired ion and eliminates interfering ions.

Figure 2 displays the idealized schematics of SRM analysis on QQQ MS. The co-eluting analytes that enter the first quadrupole are filtered based on predefined *m*/*z* values and enter the second quadrupole for collision-induced dissociation. The resulting fragment ions are then filtered by the third quadrupole passing the preset *m*/*z* values for the desired fragment ions. The two stages of mass filtering in SRM and its targeted nature lead to an increased sensitivity by one or two orders of magnitude compared with usual full scan methods. It is worthy mentioning that the term ‘multiple reaction monitoring’ (MRM) has been used to describe parallel acquisition of SRM for measurement of several target ions. However, to avoid ambiguity between the number of transitions monitored and number of stages used in the mass spectrometry analysis (MS ^*n*^), its use is deprecated by IUPAC [7].

A prototypical SRM experiment consists of three major steps. First, a list of candidate proteins is determined. The list of proteins of interest is determined based on previous knowledge from discovery studies and the scientific literature. The available information about the potentially relevant proteins (e.g., Human Protein Atlas) can also be employed in this step. In the next stage, for each candidate protein, a set of proteotypic peptides (PTPs) should be identified and targeted to determine the presence of the protein and to quantify it. PTPs of a specific protein should be able to uniquely identify that protein or one of its isoforms as well as have a good ionization efficiency. Moreover, their mass-to-charge ratio should be in the mass range of the MS instrument. Besides these general characteristics, in a quantitative experimental workflow, PTPs should be fully recovered in the sample preparation and also present good chromatographic behavior to reduce the chemical background [8]. Furthermore, post-translational and chemically induced modifications of the peptides should be taken into account. These types of peptide modifications are described in more detail in the next section, where they form a part of the model for the SRM process. Along with experimental methods, computational tools are also used to select MS-observable peptides for proteins. In the third step, for each selected peptide, the fragment ions that can unambiguously represent the targeted peptide from others should be identified. Based upon the experiments on the QQQ instrument or data from previously done shotgun experiments, two to four fragment ions are selected for each PTP. For example, being integrated with PeptideAtlas [9], TIQAM [10] can be used in this step [11].

Determination of the pairs of *m*/*z* values for the first and third quadrupoles is referred to as the selection of a *transition*[12]. The selection of transitions are of high importance for reaching high quantification accuracy and different factors such as ionization and fragmentation conditions should be taken into account. Fragmentation conditions and specially the distribution of fragment ion intensities depends on the type of instrument and the operating parameters. In the QQQ system, singly charged y-type ions are the predominant type of fragments generated by CID in a linear collision cell, as b-type ions and doubly charged fragments are significantly less stable than their y-type N-terminal counterparts [12],[13]. On the other hand, tryptic peptide ions are predominantly doubly or triply charged with one charge at each terminus. Therefore, the single-charge fragments will generally have a larger *m*/*z* value than the precursor value. On the other hand, single-charged chemical background will produce fragments with smaller *m*/*z* than the precursor. Therefore, the selection of transitions for which fragments have larger *m*/*z* than the precursor is essential for transition selectivity and high signal-to-noise ratios [12].

**Figure 3:**
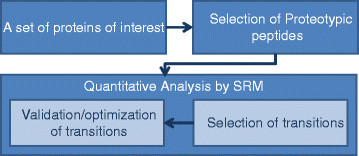
**Workflow of an SRM experiment.** First, a set of proteins of interest are determined for a specific study. Then, for each protein, some proteotypic peptides are found. In the next step, for each PTP, those fragments that are able to discriminate the peptide from others are found. The transitions (pairs of *m*/*z* values for precursor/fragment ions) are then validated to decrease the effect of unspecific signals.

In spite of the two narrow filtering stages in SRM, the selected transitions may not be specific for the peptide of interest in a complex sample. This lack of specificity can result in false quantification values for the targeted peptide. Several methods are used to validate selected transitions before using them in SRM. Spiking heavy isotope-labeled peptides to the sample, which match the sequence of the target peptide, can help in distinguishing the effect of unspecific signals. However, the cost of using heavy labeled peptides is high for quantification of large number of proteins, and usually other methods (e.g., SRM-triggered MS/MS scanning) are used, but those are unable to validate the transitions for low-abundance proteins in the detection limit of SRM [12]. Figure 3 summarizes the main steps in an SRM experiment.

## 2 Methods

In spite of the widespread application of SRM in the protein biomarker validation process, there is little work on the integration of the different modules in SRM workflow, and their systematic study to assess the impact of different parameters on the overall biomarker validation pipeline. A model-based approach toward the SRM experiment will help us to have a better understanding of the characteristics of the different modules of the SRM-based biomarker validation process. Here, the SRM pipeline is modeled as a noisy channel affecting the underlying protein abundance signal; a model for the noise channel is proposed and used to analyze the effect of different parameters and experimental settings on the final performance of the SRM-based biomarker validation pipeline and the ability of SRM to detect true biomarkers among a set of candidate ones. Although the aim of the SRM model proposed here is not to determine the exact value of each parameter, it will be useful in providing a systematic view towards studying the individual components of the SRM experiment.

### 2.1 Protein mixture model

The first major component of the model is the *protein mixture model*. This part models the abundance of the proteins in the actual SRM experiment. *Marker* and *non-marker* proteins, as well as low-abundance and high-abundance proteins, are modeled in this part. The list of candidate biomarkers in the biomarker validation stage enters the SRM pipeline as described in the previous section. As mentioned previously, there are different sources of error in the SRM workflow that result in false quantification values for the protein abundance. The situation is exacerbated when dealing with low-abundance protein biomarkers. Background high-abundance proteins, inefficiency of peptide ionization, chemically induced modification, and transition noise are the most widely quoted sources of error in SRM experiments [4],[8],[12].

In a typical experiment, the total set of samples are divided into two sample classes (e.g., *control* vs. *treatment*). There are a total number of $N_{a}^{\text{pr}}$ proteins in the mixture, among which there are $N_{c}^{\text{pr}}$ candidate proteins going through the validation stage $\left(N_{a}^{\text{pr}}>N_{c}^{\text{pr}}\right)$. Based on the observations reported in [14], the protein concentration in the pooled sample can be modeled by a Gamma distribution [15].

1 $$\eta_{i}∼\text{Gamma}(t,\theta ),\;\;i\in \left\{1,2,\ldots ,N_{a}^{\text{pr}}\right\}$$

where *t* and *θ* are shape and scale parameters, and as an example, *t*=2 and *θ*=1,000 present a realistic model with dynamic range of approximately 4 orders of magnitude.

As mentioned in the ‘Introduction’ section, many of the high-abundance protein bio-markers are already found by shotgun experiments and the focus of the SRM experiment is on validation of low-abundance candidate biomarkers. In order to model the concept of low-abundance and high-abundance proteins, we use two different Gamma distributed concentration models. For all the $N_{a}^{\text{pr}}$ proteins, and $i\in \left\{1,2,\ldots ,N_{a}^{\text{pr}}\right\}$,

2 $$\;\eta_{i}∼\;\left\{\;\begin{array}{cc} \text{Gamma}(t_{c},\theta_{c}), & \;\;i\in \;\left\{1,2,\ldots ,N_{c}^{\text{pr}}\right\}\\ \text{Gamma}(t_{a},\theta_{a}), & \;\;i\in \;\left\{(N_{c}^{\text{pr}}\;+\;1),(N_{c}^{\text{pr}}\;+\;2),\ldots ,N_{a}^{\text{pr}}\right\} \end{array}\right.$$

where *t*_*c*_, *θ*_*c*_, *t*_*a*_, and *θ*_*a*_ are the shape and scale parameters for the candidate list and background proteins, respectively. This reflects the nature of a real SRM experiment where the goal is to validate a set of low-abundance biomarkers among a complex set of high-abundance ones. We denote the number of true biomarkers in the set of $N_{c}^{\text{pr}}$ candidate list and $N_{a}^{\text{pr}}$ all proteins in the list by $N_{c}^{m}$ and $N_{a}^{m}$, respectively. The values of *t*_*c*_, *θ*_*c*_, *t*_*a*_, and *θ*_*a*_ are given in Table 1.

**Table 1:** **Parameter settings in simulation of biomarker validation model**

Parameter	Defaults value
Number of classes	2
Sample size	*n*=80
Block size	*b*=5
Block correlation	*ρ*=0.8
Fold change	*h*=2, *a*_*i*_ ∼*U* *n* *i* *f*(1,2)
Modification noise	*α*_pm_=0.03,*β*_pm_=3.6
Peptide efficiency factor	*α*_pe_=0.5,*e*_*i*_ ∼*U*(0.5,1)
Gamma parameters	*t*_*c*_=2,*θ*_*c*_=100,*t*_*a*_=5,*θ*_*a*_=10*e* 6
Purification	*β*_*γ*_=10*e*−6
Protein mixture	$N_{a}^{\text{pr}}=250,N_{c}^{\text{pr}}=40$
Ranking power	*d*=2, *r*=0.01

Biomarkers are proteins in the sample for which the expression level in the treatment and control sample differ significantly. The difference between markers and non-markers in the expression level can be modeled by fold change [15]:

3 $$f_{i}=\left\{\begin{array}{ll} a_{i}, & \;\text{if protein}\;\;\text{i}\;\;\text{is over-expressed}\\ \frac{1}{a_{i}}, & \;\text{if protein}\;\;\text{i}\;\;\text{is under-expressed}\\ 1, & \;\text{otherwise} \end{array}\right.$$

where the fold change parameter, *a*_*i*_, is uniformly distributed in [1, *h*], *h*>1. This results in a distribution that is approximately log-normal for the fold change itself [16],[17]. The value of *h* used in the simulations is specified in Table 1.

The sample variation of proteins in the mixture is modeled by a Gaussian distribution as proposed in [18], where a block model is used for the covariance matrix. The following multivariate Gaussian is used to model the concentration of the protein $i\in \left\{1,2,\ldots ,N_{a}^{\text{pr}}\right\}$ in class *j*∈{0,1} and the interaction among all the proteins in the sample:

4 $$\;C_{\text{ij}}^{\text{pr}}∼\left\{\begin{array}{ll} N\;\left(\left[\eta_{1},\eta_{2},\ldots ,\eta_{N_{a}^{\text{pr}}}\right],\Sigma \right), & \;j\in \text{class 0}\\ N\;\left(\left[f_{1}\eta_{1},f_{2}\eta_{2},\ldots ,f_{N_{a}^{\text{pr}}}\eta_{N_{a}^{\text{pr}}}\right],\Sigma \right), & \;j\in \text{class 1} \end{array}\right.$$

The covariance matrix *Σ* has a block structure, such that

5 $$\begin{array}{l} \Sigma ={\left[\sigma_{\text{ij}}^{2}\right]}_{N_{a}^{\text{pr}}\times N_{a}^{\text{pr}}}\\ \sigma_{\text{ij}}^{2}=\sigma_{\text{ii}}\sigma_{\text{jj}}\lambda_{\text{ij}}\\ \sigma_{\text{ii}}=\phi_{i}\times \eta_{i} \end{array}$$

where the constant *ϕ* is the coefficient of variation and the correlation matrix *Λ* is defined as follows:

6 $$\begin{array}{l} \Lambda =[\;\lambda_{\text{ij}}]=\left[\begin{array}{llll} R_{\rho } & 0 & \cdots & 0\\ 0 & R_{\rho } & \cdots & 0\\ \vdots & \vdots & \ddots & \vdots \\ 0 & 0 & \cdots & R_{\rho } \end{array}\right], \end{array}$$

where *R*_*ρ*_ is a *b*×*b* matrix with 1’s on the diagonal and *ρ*’s elsewhere. The block-based structure of the covariance matrix represents the real interaction among the proteins. The proteins in each block (e.g., proteins within a pathway) are correlated, while there is no interaction among the proteins of different blocks [18]. The correlation *ρ* and block size *b* control the level of interaction among the proteins and their corresponding value used in simulations are specified in Table 1.

### 2.2 Sample complexity and purification

Many of the biomarkers with high abundance have already been found, and the main interest in SRM-based biomarker validation process is in the quantification of low-abundance proteins. In biological samples, there is a wide dynamic range in protein abundance (> 10^10^), which is much larger than the dynamic range of many MS instruments. For example, while interleukin has very low abundance, albumin makes up more than 50% (about 60%) of human plasma protein (30 to 50 g/L for albumin compared to below 100 pg/L for interleukin) [19].

Presence of high-abundance proteins interfering with the low-abundance ones biases the detection and quantification of biomarkers in complex samples. For example, due to the suppression of their ionization by high-abundance proteins, low-abundance proteins escape detection. This makes purification and removal of high abundant proteins an important stage of biomarker validation workflow. Purification removes background noise in the data, i.e., the nonspecific contributions of proteins not being evaluated as candidate markers [2],[20]. There are different commercial and noncommercial options for the enrichment of samples for low-abundance proteins, and the amount of energy that is put in this step greatly affects the overall performance of biomarker identification in the SRM process. For example, albumin precipitation, size exclusion, and immuno-depletion are strategies that have been developed to eliminate some of the most abundant proteins from blood serum. As an specific example, Seppro®; IgY12 (Sigma-Aldrich, St. Louis, Missouri 63103, USA) removes 12 high-abundance proteins from human biological fluids such as serum, plasma, and cerebral spinal fluid (CSF) [21].

In this paper, we model purification by removing a set of high-abundance proteins from the protein mixture model. The parameter *p*_p_ controls the purification in the model by indicating the percentage of high-abundance proteins that are successfully removed. Denoting the set of proteins selected for purification by $G_{p}$, we have the following:

7 $${Ĉ}_{\text{ij}}^{\text{pr}}=\left\{\begin{array}{ll} \gamma_{i}C_{\text{ij}}^{\text{pr}}, & \;\text{if protein}\text{i}\in G_{p}\\ C_{\text{ij}}^{\text{pr}}, & \;\text{otherwise} \end{array}\right.$$

where *γ*_*i*_∼*U*(0,*β*_*γ*_). The value used for *β*_*γ*_ (0 <*β*_*γ*_ <<1) in the simulations is given in Table 1.

### 2.3 Peptide mixture model

As mentioned in the ‘Introduction’ section, for each protein in the list of candidate biomarkers, a set of PTPs is identified and targeted to determine the presence of the protein and to quantify it. PTPs should uniquely identify the proteins, have good ionization efficiency, be fully recovered during sample preparation, and also present good chromatographic behavior to reduce the chemical background [8].

The molar concentration of $C_{i}^{\text{pp}}$ of peptide *i* in each sample, in class *j*, is given by

8 $$C_{\text{ij}}^{\text{pp}}=\underset{k\in \Omega_{i}}{\sum }{Ĉ}_{\text{kj}}^{\text{pr}},\;i\in \left\{1,2,\ldots ,N_{c}^{\text{pp}}\right\},j\in \{0,1\}$$

where *Ω*_*i*_ is the set of all proteins sharing peptide species *i* and $N_{c}^{\text{pp}}$ is the number of peptides. In an usual SRM experiment, for each protein, 1 to 2 PTPs are used. Denoting the number of peptides per protein by *N*_pp_, then $N_{c}^{\text{pp}}$ is equal to $N_{\text{pp}}\times N_{a}^{\text{pr}}$. In the results reported in this paper, we set *N*_pp_=2. In the ideal case, the cardinality of the set *Ω*_*i*_ is 1, that is $C_{i}^{\text{pp}}$, the concentration of peptide *i*, is related to only one protein. Equation (8) can be rewritten as following:

9 $$C_{\text{ij}}^{\text{pp}}=\overset{N_{a}^{\text{pr}}}{\underset{k=1}{\sum }}\xi_{\text{ik}}{Ĉ}_{\text{kj}}^{\text{pr}},\;i\in \left\{1,2,\ldots ,N_{c}^{\text{pp}}\right\},j\in \{0,1\}$$

where for $i\in \left\{1,2,\ldots ,N_{c}^{\text{pp}}\right\}$ and $k\in \left\{1,2,\ldots ,N_{a}^{\text{pr}}\right\}$, *ξ*_*ik*_ is as follows:

10 $$\xi_{\text{ik}}=\left\{\begin{array}{ll} 1, & \text{protein}\;\text{k}\;\;\text{has peptide}\text{j}\\ 0, & \text{otherwise} \end{array}\right.\;.$$

In an ideal SRM experiment, each peptide is specific to one protein and then the peptide-protein relation matrix $\Xi =\;{\left[\;\xi_{\text{ik}}\right]}_{N_{c}^{\text{pp}}\times N_{a}^{\text{pr}}}$ has only one element equal to 1 in each row. In real SRM experiments, the complexity of the sample increases the possibility of having target peptides as a part of other proteins. To model this fact, we define *s*_*i*_, the specificity of the *i* th PTP, as

11 $$s_{i}=1-P\;\left(\left|\Xi_{i}^{1}\right|\neq 1\right)$$

where |*S*| shows the cardinality (the number of elements) of the set *S* and $\Xi_{i}^{1}$ is the set of nonzero elements of the *i* th row of PTP-protein relation matrix *Ξ*. A peptide among the list of PTPs is called *specific* if its share in the sample is created by only its parent target protein. The specificity *s*_*i*_ of a specific PTP is then equal to 1. However, in real SRM experiments, this idealized situation does not occur and for some of the proteotypic peptides, the specificity will be less than 1.

There are many factors that should be considered in choosing the PTPs for each protein. For example, for each PTP, MS properties, uniqueness, and chemical behavior should be taken into account [12]. Increasing the number of proteins exacerbates the problem of finding PTPs that are specific to the target proteins and comply with other PTP selection criteria. On the other hand, we are not interested in the exact specificity value of each PTP but rather want to observe the general effect of PTP specificity on the overall performance of biomarker validation process by SRM experiment. We thus define *s* as the average specificity over all peptides and study its effect on the identification of low-abundance protein biomarkers.

### 2.4 Peptide ionization efficiency

The abundance of a peptide is represented by the ion abundance in MS data. The abundance of a peptide *i* in class *j* is modeled by

12 $$\mu_{\text{ij}}=\kappa \;e_{i}\;C_{\text{ij}}^{\text{pp}}\;,$$

where *e*_*i*_ is the peptide efficiency factor, similar to [22], and *κ* represents the instrument response factor, being the ratio between the ion current signal and the original analyte concentration.

The efficiency of different peptides in passing through the liquid chromatography column is mainly controlled by their hydrophobicity [2], followed by ionization efficiency, which is affected by sample complexity, peptide concentration, and characteristics such as polarity of side chains, molecular bulkiness, and so on [15],[23]. Efficiency is also affected by the destabilizing effect of some amino acids at the N-terminal end of peptides. Some methods have been proposed for the prediction of *e*_*i*_ for different peptides. However, these methods fail to address the complexity issue and dependence of the efficiency on not only the underlying peptide but also on the other peptides present [15].

This makes the prediction of *e*_*i*_ for all the peptides problematic. Here, instead of the exact value of *e*_*i*_, we are more interested in its effect on the overall performance of the SRM experiment. In the ideal case, *e*_*i*_ is 1 for all peptides. A model based on the uniform distribution *U*(*α*_pe_,1) models the variation of the peptide efficiency. The parameter *α*_pe_ controls the dispersion of the ionization efficiency and in the ‘Results and discussion’ section, we analyze the model over a wide range to observe the effect of this parameter on the performance of the biomarker validation process.

### 2.5 Transition

In a complex sample, a particular precursor/fragment combination may not be specific to a targeted peptide, and other peptides with precursor/fragment ion pairs of similar masses might create unspecific signals. In the case that SRM is used to target low-abundant peptides, such unspecific signals, might still be well above the detection limit and might be easily mistaken as being derived from the targeted peptide and thus lead to misquantifications [12]. Validation methods are used to ensure that the origin of the quantified signal is the targeted peptide. SRM-triggered MS/MS scanning is the method of choice in different studies. However, this method is challenging when used for the most low-abundance peptides [24]. Spiking heavy-isotope-labeled peptides into the sample is an alternative for the use of SRM-triggered MS/MS. But the costs of such method can be very high for projects targeting a large number of proteins. In addition, the application of stable isotopes is limited by the resolution of the quadrupole as isotope labeling should introduce a sufficiently large mass difference between precursor and fragment ions [12]. Using smaller mass differences in isotope-labeling requires a higher resolution for the quadrupole, which in turn decreases the sensitivity. Low resolution has been reported in many papers as a source of error for SRM experiments using triple quadrupole mass spectrometers in complex samples [4].

The effect of transitions from background high-abundance peptides is considered as a significant source of error in quantification of the low-abundant peptides. Unspecific signals are created from other peptides with ion pairs of similar masses with the targeted peptide. By increasing the measured abundance of the targeted peptide, the unspecific signals create misquantification. Therefore, the noise is always positive. The exponential distribution is a simple and adequate choice to model this kind of unipolar additive noise

13 $$\zeta_{\text{ij}}=\mu_{\text{ij}}+\varepsilon_{\text{ij}}^{t}\;,$$

where

14 $$\varepsilon_{\text{ij}}^{t}∼\exp(\mu_{\text{tran}}\mu_{\text{ij}})\;.$$

### 2.6 Peptide modification

Standard sources of error, including variation in experimental conditions, instrument variance, and thermal noise, can affect the accuracy of quantitative MS experiments. Besides these general factors, peptide modification is reported as one of the important causes of misquantification in SRM experiments [12].

Some peptides contain amino acids with high propensity to chemical modifications and can bias the quantification. Cysteine alkylation, methionine oxidation, asparagine deamidation, and N-terminal cyclization of glutamic are some of the chemically induced modification of peptides [8]. Oxidation, for example, is reported to inversely affect the performance of MS experiments for quantification of peptides [25]. Since a part of the targeted peptide is converted into the modified form during the process, chemically induced modification is reported to be a potential source of error in quantitative MS experiments [8],[12].

The Gaussian distribution is the standard model for the cumulative effect of independent additive disturbances (distinct noise sources). In [26], a Gaussian noise model with quadratic dependence of the variance on the expected abundance of peptide is used to model the overall effect of different noise sources affecting the actual abundance of a peptide in LC-MS. Likewise, we propose to use the Gaussian noise to model the effect of peptide modification as well as the other sources of error with significant impact on modifying the actual abundance of the peptide in SRM (LC-MS-MS). We have

15 $$\nu_{\text{ij}}=\zeta_{\text{ij}}+\varepsilon_{\text{ij}}^{m}\;,$$

where

16 $$\varepsilon_{\text{ij}}^{m}∼N\;\left(0,\alpha_{\text{pm}}\nu_{\text{ij}}^{2}+\beta_{\text{pm}}\nu_{\text{ij}}\right)\;.$$

The two parameters *α*_pm_ and *β*_pm_ control the severity of the noise. In [26], a replication analysis is proposed to estimate the values of these two parameters. The values of *α*_pm_ and *β*_pm_ used in simulations are specified in Table 1. Having fixed *β*_pm_, we will investigate the effect of *α*_pm_ on the performance of the biomarker validation in the next section.

## 3 Results and discussion

**Figure 4:**
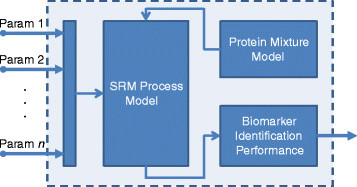
**The entire simulation process.** The protein abundance mixture data enters the SRM process and is affected by different noise sources in different levels of the process. The noisy data enters the biomarker validation block, where the ranking power and true positive rate are used to measure the performance of the overall biomarker validation process.

The previous modeling strategy is used to analyze the performance of biomarker validation workflow using SRM experiments, using different model parameter settings. Figure 4 displays the simulation process. The list of candidate biomarkers generated based on the protein mixture model is the input of the SRM pipeline. In different stages of this process, the protein mixture data is affected by different noise sources depending on the experiment setting. Then, the output of the SRM process enters the validation block. Ranking power [27] and percentage of true biomarkers are used as the metrics to assess the performance of the biomarker identification process. The model parameters are changed during the simulation and for each parameter setting the average performance is found. The ranking power is described in the next section.

### 3.1 Experimental setup

We perform a total of 5,000 Monte Carlo runs in this experimental study, using the parameter settings given in Table 1, and compute average performance metrics over all the runs.

The performance metrics used to evaluate SRM performance are the percentage of peptides correctly identified and the *ranking power*[27]. The former is computed by applying the *t*-test as a feature selection method to find the best discriminant set of features, and computing the ratio of true biomarkers detected in that list. The latter defines a measure of goodness based on how close the estimate-based feature sets are to optimality. Let *A*_best_ be the best feature set relative to the feature-label distribution, *ε*_0_ be the true error of the classifier for *A*_best_ designed on the sample, and *A*_(1)_,*A*_(2)_,…,*A*_(*m*)_ be a list of feature sets ordered by the classification errors *ε*_1_,*ε*_2_,…,*ε*_*m*_, sorted from lowest to highest. The ranking power of the list is defined by

17 $$\Delta_{D,d}^{n,r}=P(\varepsilon_{1}-\varepsilon_{0}<r)\;,$$

for *r*>0. The ranking power gives the probability that at least one feature set in the list has error within *r* of the best feature set. The closer $\Delta_{D,d}^{n,r}$ is to 1, the better the performance is (as long as *m* is small; here, *m*=10 is used).

The pseudocode for computing the power rank is described in Algorithm 1.

### 3.2 Effect of purification

**Figure 5:**
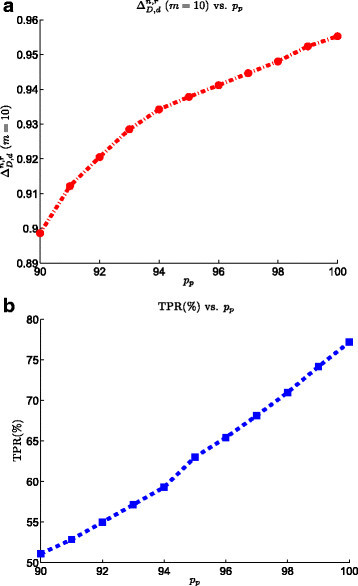
**Effect of purification on the the SRM model on the performance of the biomarker validation pipeline.** **(a)**$\Delta_{D,d}^{n,r}$ at list size *m*=10 vs. purification. **(b)** TPR vs. purification.

Figure 5 displays the effect of purification on the performance of the SRM biomarker validation process. We can see that increasing the purification factor from 90*%* to 99*%* increases the ranking power by 7*%*. Increasing the purity from 90*%* to 99*%* translates into the increase of TPR from 50*%* to 80*%*. Although our purpose is not to focus on the exact value of each parameter in the model, the results show how purification is an important step in the SRM experiment. This confirms the fact that purification strategies, such as albumin precipitation, size exclusion, and immuno-depletion, directly control the accuracy of the SRM-based biomarker validation.

### 3.3 Effect of peptide specificity

**Figure 6:**
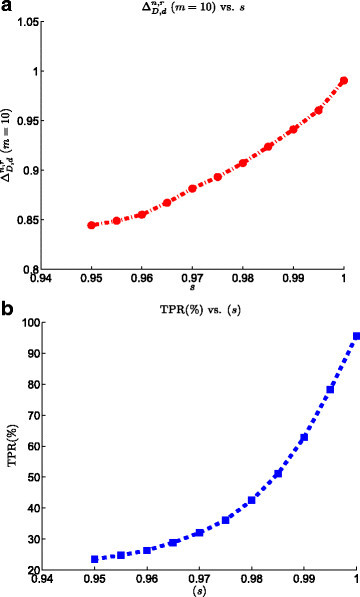
**Effect of peptide specificity on the SRM model on the performance of the biomarker validation pipeline.** **(a)**$\Delta_{D,d}^{n,r}$ at list size *m*=10 vs. peptide specificity. **(b)** TPR vs. peptide specificity.

Figure 6 shows the effect of peptide specificity on the performance of SRM biomarker validation process. The results show that a very small amount of decrease in the specificity factor can bias the quantification of the low-abundance proteins to a great extent. For example, decreasing the specificity from 1 to 0.95 decreases the TPR by about 75*%*. These results indicate the importance of the selection of proper set of proteotypic peptides emphasizing on the fact that PTPs of a specific protein should be able to uniquely identify the protein (being specific peptides).

### 3.4 Effect of peptide efficiency

**Figure 7:**
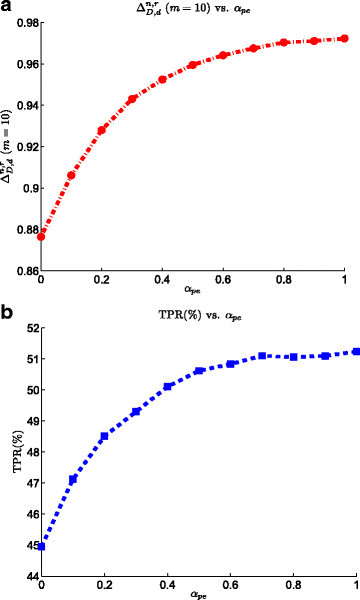
**Effect of peptide efficiency on the SRM model on the performance of the biomarker validation pipeline.** **(a)**$\Delta_{D,d}^{n,r}$ at list size *m*=10 vs. peptide efficiency. **(b)** TPR vs. peptide efficiency.

Although the exact distribution of the peptide efficiency is not known, observing its effect on the overall performance of the biomarker validation process provides us with a good insight into the effect of this parameter on the SRM experiment. This effect can be seen in Figure 7. The variation of peptide efficiency factor, *α*_pe_ (the lower bound of *e*_*i*_), in the interval [ 0,1] changes the TPR by 6*%*, increasing it from 45*%* at *α*_pe_=0 to 51*%* at *α*_pe_=1. Based on the ranking power plot, we observe a similar trend: $\Delta_{D,d}^{n,r}$ increases from 0.88 to 0.97 by increasing the peptide efficiency factor from 0 to 1. These results agree with our expectations as the increase of the peptide efficiency reduces the transmission loss.

### 3.5 Effect of transition noise

**Figure 8:**
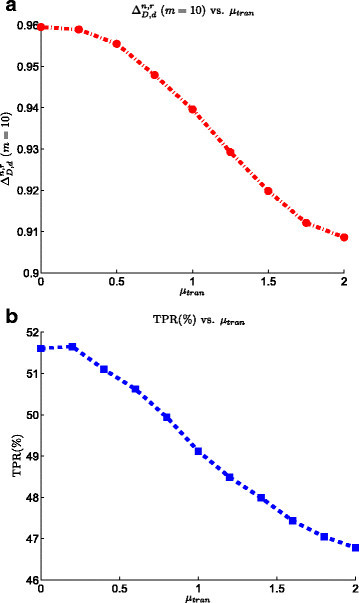
**Effect of transition noise on the the SRM model on the performance of the biomarker validation pipeline.** **(a)**$\Delta_{D,d}^{n,r}$ at list size *m*=10 vs. transition noise. **(b)** TPR vs. transition noise.

Figure 8 shows the effect of transition noise on the performance of SRM biomarker validation process. Both the ranking power and TPR curves show that an increase of the transition noise decreases the overall performance of the biomarker validation. For example, the ranking power is 0.96 when the effect of this noise is set to zero. However, by increasing the noise factor to 2, $\Delta_{D,d}^{n,r}$ reduces to 0.91. We observe a similar behavior, looking at TPR curve, where the rate decreases by 7*%* as the transition noise increases. This emphasizes the importance of applying the proper methods for validation of the transitions to increase the confidence on the origin of the quantified signal. Based on the experiment constraints, methods such as SRM-triggered MS/MS scanning and spiking of heavy-isotope-labeled peptides should be used to prevent the contribution of unspecific signals in the quantification of the proteins of interest.

### 3.6 Effect of modification

**Figure 9:**
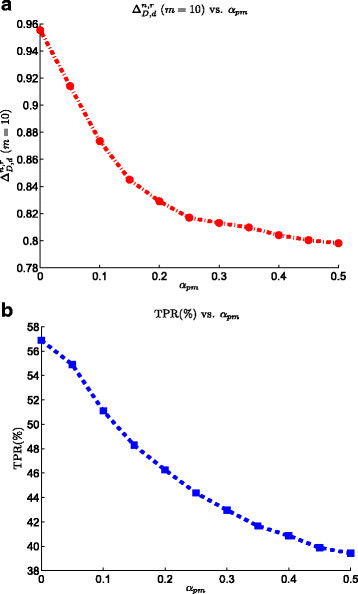
**Effect of modification noise on the the SRM model on the performance of the biomarker validation pipeline.** **(a)**$\Delta_{D,d}^{n,r}$ at list size *m*=10 vs. modification noise. **(b)** TPR vs. modification noise.

Figure 9 displays the effect of modification noise on the performance of the SRM biomarker validation process. Increasing the modification noise factor *α*_pm_ from 0 to 0.5 reduces the TPR value by 17*%*. On the other hand, the ranking power plot behaves the same by decreasing *α*_pm_ from 0.96 to 0.8. Decreasing the modification noise from 0.2 to 0 dramatically increases the ranking power value, emphasizing the fact that reduction of this source of error in quantification of the low-abundance biomarkers is crucial for a successful SRM experiment. This also shows that one should avoid using peptides with high tendency for chemical modifications in the list of PTPs.

### 3.7 Effect of sample size

**Figure 10:**
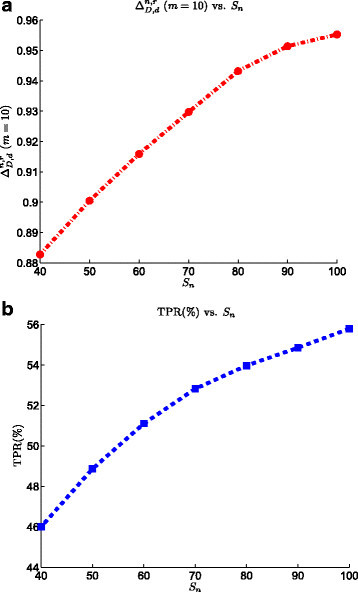
**Effect of sample size on the the SRM model on the performance of the biomarker validation pipeline.** **(a)**$\Delta_{D,d}^{n,r}$ at list size *m*=10 vs. sample size. **(b)** TPR vs. sample size.

Compared to the discovery stage of biomarker development, where thousands of analytes are measured, a validation experiment deals with the quantification of a limited list of analytes, meaning that the sample size requirement is less demanding. However, the time and cost of the experiment as well as the challenges of finding patients with correct demographics for the disease of interest, with proper medical history and lifestyle, still restricts the number of samples in a biomarker validation experiment to the ‘small-sample’ region [28]. Observing the effect of the number of samples on the performance of the biomarker validation process will be beneficial to the selection of the right amount of replicates considering the limitations on the time and cost of the experiment. Figure 10 shows the effect of sample size on the performance of SRM biomarker validation process. Both TPR and ranking power plots show that these two performance indices are greatly affected by the increase of the sample size. Increase of the sample size from 40 to 100 results in 10*%* increase in the TPR value. The similar change in the sample size translates into the increase of ranking power value by 0.07.

### 3.8 Summary

General facts can be gleaned from the results reported above in the paper on the relative importance of each parameter to the sensitivity of biomarker validation performance using the QQQ-based SRM system.

Purification critically increases the efficiency of the whole pipeline by reducing the background high-abundance proteins.

On the other hand, peptide ionization efficiency also plays an important role in the success of biomarker validation experiment.

A high value of modification noise can greatly compromise the performance of the system, as measured by the decreases of the TPR and ranking power value.

Likewise, a decrease of peptide specificity reduces the TPR and ranking power to a great extent.

The results emphasize the importance of the correct selection of peptides in an SRM experiment. If the selected peptides are not unique to the targeted protein, it is hard to have high-precision quantification of the abundance of the targeted peptides, which will show itself in the unsuccessful protein validation results. An additional factor is of course sample size, which not surprisingly showed a clear effect on the performance of the biomarker discovery pipeline.

## 4 Conclusions

In this paper, the key components of the typical SRM-based biomarker validation workflow were reviewed, modeled, and analyzed. Based on the synthetic data, the process was simulated and the effect of different parameter setting on the performance was studied. Ranking power and the TPR were used as two different metrics to assess the performance of the biomarker validation process as a function of the parameters of the model. The goal of this study was not the determination of the exact value of each parameter for reaching a given performance value but rather to investigate the effect of the different parameters, namely, sample purification, peptide ionization efficiency, peptide specificity, modification noise, and sample size, on the overall performance of the SRM experiment utilized for biomarker validation.

The model presented here can not only be utilized to observe the effect of different instrument and experimental settings on biomarker validation by SRM but also could be useful for experimental design, providing an insight on the working range of the important parameters of the SRM pipeline. It creates the required infrastructure for studying the inverse problem, where one can use the model to set the parameters of the entire experiment to reach the highest performance considering technical, experimental and financial constraints. Also, the model has the advantage of being flexible to future possible extension in order to include more detailed modules of the SRM pipeline.

## Authors’ original submitted files for images

Below are the links to the authors’ original submitted files for images. Authors’ original file for figure 1 Authors’ original file for figure 2 Authors’ original file for figure 3 Authors’ original file for figure 4 Authors’ original file for figure 5 Authors’ original file for figure 6 Authors’ original file for figure 7 Authors’ original file for figure 8 Authors’ original file for figure 9 Authors’ original file for figure 10

## Abbreviations

QQQ: triple quadrupole
MS: mass spectrometry
SRM: selected reaction monitoring
PSA: prostate-specific antigen
CID: collision-induced dissociation
PTP: proteotypic peptides
TPR: true positive rate

## References

[1] Aebersold R, Mann M (2003). Mass spectrometry-based proteomics. Nature.

[2] Dass C (2007). Fundamentals of Contemporary Mass Spectrometry.

[3] de Hoffmann E (1996). Tandem mass spectrometry: a primer. J. Mass. Spectrom.

[4] Kitteringham NR, Jenkins RE, Lane CS, Elliott VL, Park BK (2009). Multiple reaction monitoring for quantitative biomarker analysis in proteomics and metabolomics. J. Chromatogr. B.

[5] Zakett D, Flynn R, Cooks R (1978). Chlorine isotope effects in mass spectrometry by multiple reaction monitoring. J. Phys. Chem.

[6] Baty J, Robinson P (1977). Single and multiple ion recording techniques for the analysis of diphenylhydantoin and its major metabolite in plasma. Biol. Mass Spectrom.

[7] Murray KK, Boyd RK, Eberlin MN, Langley GJ, Li L, Naito Y (2013). Standard definitions of terms relating to mass spectrometry (IUPAC recommendations 2013), Analytical Chemistry Division. Pure Appl. Chem.

[8] Gallien S, Duriez E, Domon B (2011). Selected reaction monitoring applied to proteomics. J. Mass Spectrom.

[9] Deutsch EW, Lam H, Aebersold R (2008). PeptideAtlas: a resource for target selection for emerging targeted proteomics workflows. EMBO Rep.

[10] Lange V, Malmström JA, Didion J, King NL, Johansson BP, Schäfer J, Rameseder J, Wong CH, Deutsch EW, Brusniak MY, Bühlmann P, Björck L, Domon B, Aebersold R (2008). Targeted quantitative analysis of streptococcus pyogenes virulence factors by multiple reaction monitoring. Mol. Cell. Proteomics.

[11] Malmström L, Malmström J, Selevsek N, Rosenberger G, Aebersold R (2012). Automated workflow for large-scale selected reaction monitoring experiments. J. Proteome Res.

[12] Lange V, Picotti P, Domon B, Aebersold R (2008). Selected reaction monitoring for quantitative proteomics: a tutorial. Mol. Syst. Biol.

[13] Lau KW, Hart SR, Lynch JA, Wong SC, Hubbard SJ, Gaskell SJ (2009). Observations on the detection of b-and y-type ions in the collisionally activated decomposition spectra of protonated peptides. Rapid Commun. Mass. Spectrom.

[14] Taniguchi Y, Choi PJ, Li GW, Chen H, Babu M, Hearn J, Emili A, Xie XS (2010). Quantifying *E. coli* proteome and transcriptome with single-molecule sensitivity in single cells. Science.

[15] Sun Y, Neto Braga-U, Dougherty ER (2012). A systematic model of the LC-MS proteomics pipeline. BMC Genomics.

[16] Furusawa C, Suzuki T, Kashiwagi A, Yomo T, Kaneko K (2005). Ubiquity of log-normal distributions in intra-cellular reaction dynamics. Biophysics.

[17] Furusawa C, Kaneko K (2007). Universal statistics for chemical abundances in a reproducing cell. J. Korean Phys. Soc.

[18] Hua J, Tembe WD, Dougherty ER (2009). Performance of feature-selection methods in the classification of high-dimension data. Pattern Recognit.

[19] Anderson NL, Anderson NG (2002). The human plasma proteome history, character, and diagnostic prospects. Mol. Cell. Proteomics.

[20] Xu X, Veenstra TD (2008). Analysis of biofluids for biomarker research. Proteomics-Clinical Appl.

[21] Bandow JE (2010). Comparison of protein enrichment strategies for proteome analysis of plasma. Proteomics.

[22] Timm W, Scherbart A, Böcker S, Kohlbacher O, Nattkemper TW (2008). Peak intensity prediction in MALDI-TOF mass spectrometry: a machine learning study to support quantitative proteomics. BMC Bioinf.

[23] Cech NB, Enke CG (2002). Practical implications of some recent studies in electrospray ionization fundamentals. Mass Spectrom. Rev.

[24] Picotti P, Rinner O, Stallmach R, Dautel F, Farrah T, Domon B, Wenschuh H, Aebersold R (2009). High-throughput generation of selected reaction-monitoring assays for proteins and proteomes. Nat. Methods.

[25] Froelich JM, Reid GE (2008). The origin and control of ex vivo oxidative peptide modifications prior to mass spectrometry analysis. Proteomics.

[26] Anderle M, Roy S, Lin H, Becker C, Joho K (2004). Quantifying reproducibility for differential proteomics: noise analysis for protein liquid chromatography-mass spectrometry of human serum. Bioinformatics.

[27] Zhao C, Bittner ML, Chapkin RS, Dougherty ER (2010). Characterization of the effectiveness of reporting lists of small feature sets relative to the accuracy of the prior biological knowledge. Cancer Inf.

[28] Ye X, Blonder J, Veenstra TD (2009). Targeted proteomics for validation of biomarkers in clinical samples. Brief. Funct. Genomics Proteomics.

