# Supplementary figures and images for: Modeling and systematic analysis of biomarker validation using selected reaction monitoring
Source: EURASIP J Bioinform Syst Biol. 2014 Nov 15;2014:17. doi: 10.1186/s13637-014-0017-y (PMC5270363; doi:10.1186/s13637-014-0017-y)

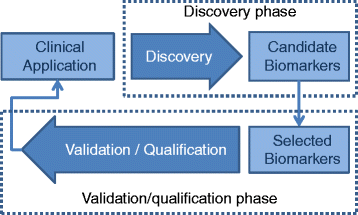

Supplement: Supplementary file 1 — Authors’ original file for figure 1 [file 13637_2014_17_MOESM1_ESM.gif]

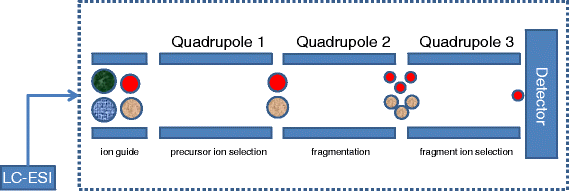

Supplement: Supplementary file 2 — Authors’ original file for figure 2 [file 13637_2014_17_MOESM2_ESM.gif]

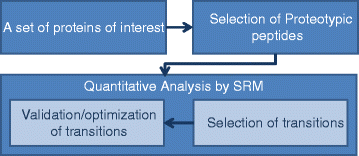

Supplement: Supplementary file 3 — Authors’ original file for figure 3 [file 13637_2014_17_MOESM3_ESM.gif]

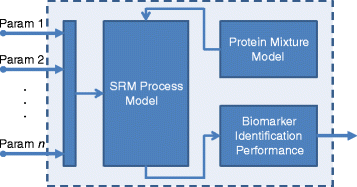

Supplement: Supplementary file 4 — Authors’ original file for figure 4 [file 13637_2014_17_MOESM4_ESM.gif]

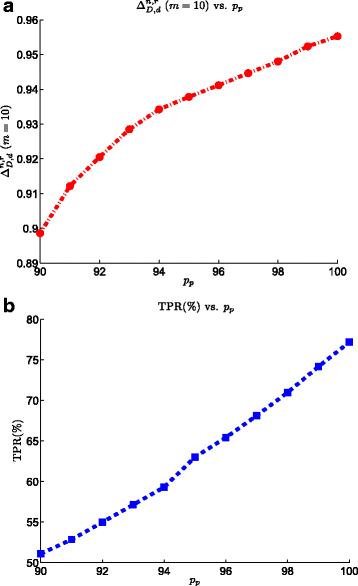

Supplement: Supplementary file 5 — Authors’ original file for figure 5 [file 13637_2014_17_MOESM5_ESM.gif]

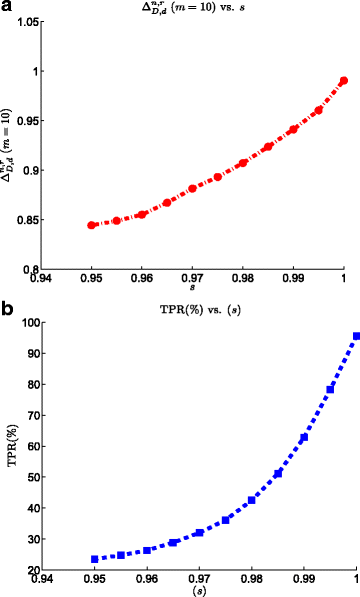

Supplement: Supplementary file 6 — Authors’ original file for figure 6 [file 13637_2014_17_MOESM6_ESM.gif]

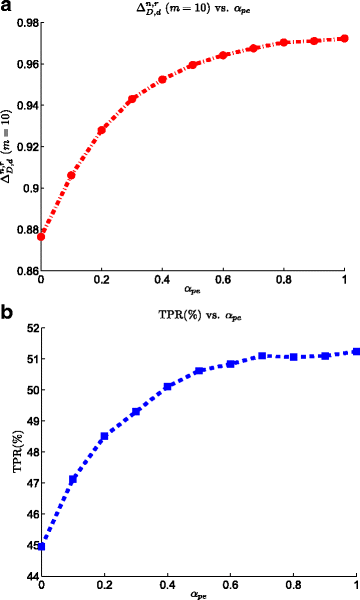

Supplement: Supplementary file 7 — Authors’ original file for figure 7 [file 13637_2014_17_MOESM7_ESM.gif]

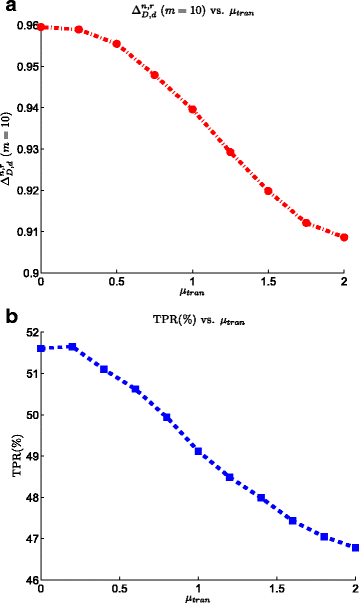

Supplement: Supplementary file 8 — Authors’ original file for figure 8 [file 13637_2014_17_MOESM8_ESM.gif]

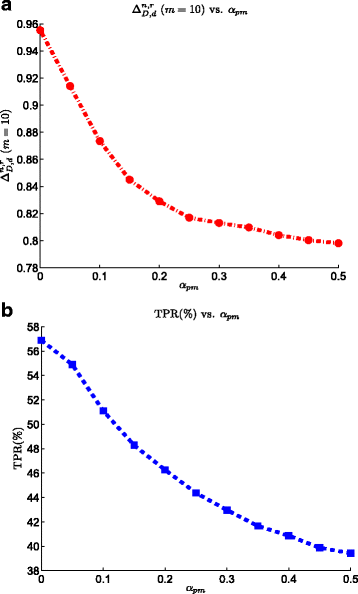

Supplement: Supplementary file 9 — Authors’ original file for figure 9 [file 13637_2014_17_MOESM9_ESM.gif]

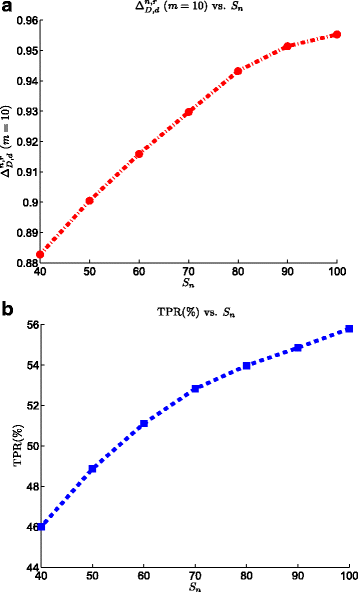

Supplement: Supplementary file 10 — Authors’ original file for figure 10 [file 13637_2014_17_MOESM10_ESM.gif]
